# *Errata:* Vol. 66, Nos. SS-1 and SS-2

**DOI:** 10.15585/mmwr.mm6603a11

**Published:** 2017-01-27

In the Surveillance Summaries “Leading Causes of Death in Nonmetropolitan and Metropolitan Areas — United States, 1999–2014” and “Reducing Potentially Excess Deaths from the Five Leading Causes of Death in the Rural United States,” an error occurred in Figure 5 and Figure 3, respectively. In the last panel of bar charts (stroke), the colors for the left-most set of bars (public health region 1) **should be reversed**. 

In addition, the following person should have been listed as the Guest Editor in the masthead and the Acknowledgments: **Robin M. Wagner, PhD, MS, *Guest Editor*.**

